# Challenging Hurdles of Current Targeting in Glioblastoma: A Focus on Immunotherapeutic Strategies

**DOI:** 10.3390/ijms22073493

**Published:** 2021-03-28

**Authors:** Vassilis Genoud, Denis Migliorini

**Affiliations:** 1 Department of Oncology, University Hospital of Geneva, 1205 Geneva, Switzerland; vassilis.genoud@hcuge.ch; 2 Center for Translational Research in Onco-Haematology, University of Geneva, 1205 Geneva, Switzerland; 3 Brain Tumor and Immune Cell Engineering Laboratory, 1005 Lausanne, Switzerland; 4 Swiss Cancer Center Léman, 1205 Geneva, Switzerland

**Keywords:** glioblastoma, immunotherapy, clinical trials

## Abstract

Glioblastoma is the most frequent primary neoplasm of the central nervous system and still suffers from very poor therapeutic impact. No clear improvements over current standard of care have been made in the last decade. For other cancers, but also for brain metastasis, which harbors a very distinct biology from glioblastoma, immunotherapy has already proven its efficacy. Efforts have been pursued to allow glioblastoma patients to benefit from these new approaches, but the road is still long for broad application. Here, we aim to review key glioblastoma immune related characteristics, current immunotherapeutic strategies being explored, their potential caveats, and future directions.

## 1. Introduction

The incidence of glioblastoma (GBM) is 7.23 cases per 100,000 patients a year, and the outcome is very poor, with a median survival of less than 2 years [1]. These numbers translate the devastating reality of current GBM clinical management and the desperate need for better therapeutics.

However, since the 2010s and the first clinical trial proving the efficacy of immunotherapy for solid tumors, the field of oncology has radically changed for some cancer indications, with unprecedented survival improvement. Still, GBM has not benefited from these novel therapies yet. Even though, in the past, the central nervous system (CNS) was considered to be immune-privileged [2], we now may characterized it to be immune-specialized. The efficacy of immunotherapy on the CNS is proven, as activity against brain metastasis, mostly from melanoma or lung cancer, has been widely described [3]. To that matter, the location of the tumor on the CNS may not be the sole limiting factor, but rather the intrinsic immuno-suppressive characteristics of GBM.

Here, we will discuss the reasons for this setback by first describing key GBM characteristics, then potential targets and specific immunotherapeutic strategies developed and tested in clinical trials. Finally, we will address future directions that should pave the way for the improvement of immunotherapy for GBM.

### 1.1. GBM Characteristics

#### 1.1.1. Molecular Alterations and Subtypes

The pathological description of GBM is described by the WHO classifications and is comprised of histological and molecular alterations [4]. GBM corresponds to grade IV, the most aggressive type of gliomas, originating from glial cells. Primary GBM initially presents as grade IV, as opposed to secondary GBM originating from lower grade tumors. Primary GBM (Isocitrate dehydrogenase (IDH) wild type) has a worse prognosis compared to secondary GBM [5]. The Cancer Genome Atlas Program (TCGA) led to a better understanding of GBM’s molecular characteristics, and revealed the presence of different molecular subtypes [6]. Despite the low clinical implications of molecular subtypes, due to the high heterogeneity of GBM tumors [7], it helped to identify common and shared genomic alterations, which helped to develop rationally designed pre-clinical models of GBM by targeting the same genomic alterations. For example, the SB28 is a syngeneic mouse model developed by targeting mutations attributed to the proneural subtype (p53, RAS and PDGF) [8] or the spontaneous model PDGF-B–driven glioma, which targets the PDGF pathway [9,10]. Deeper understanding of molecular characteristics also impacted on GBM description and diagnosis. The cIMPACT-NOW classification empowers the 2016 WHO classification with broad genomic integration [5]. Analysis of the methylome also proves its clinical implication, as it can help to reclassify tumors to the right category [11]. Mutational load is directly liked to immunotherapy as it can lead to the generation of neoantigens (NeoAg), which are known to be immunogenic [12]. Correlation between the tumor mutational burden (TMB) and the response to immunotherapy has been described, but GBM belongs to the category of tumors with low TMB [12]. Other factors, such as a low expression of antigen (Ag) presentation in MHC molecules, are described in GBM, therefore leaving more challenges to generating an effective immune response [13].

#### 1.1.2. Immune Specialization

From an immunological point of view, the CNS draws specificities in the well described cancer immunity cycle [14]. First of all, the blood–brain barrier (BBB) limits the interactions between the two compartments that can limit cell migration from blood to the CNS. Nevertheless, we now have robust data proving the T cell efflux to the CNS, particularly through the α4β1 integrin [15]. Moreover, cytokines can transiently open the BBB, such as nitric oxide (NO) or tumor necrosis factor (TNF), and GBM can also locally disrupt the BBB [16]. Still, physical factors such as the antibody (Ab) size need to be taken into account for optimal drug delivery [17], even though radiolabeled Abs are clearly shown to locate at the tumor site, such as in brain metastasis [18]. Secondly, the CNS lacks conventional draining lymphatics, but evidence points towards adapted drainage to deep cervical lymph nodes [19,20]. Conventional dendritic cells (DCs) are also missing in the brain parenchyma, which is populated by an important proportion of microglia cells that can also present antigens [21]. Still, the GBM tumor microenvironment (TME) is mostly immunosuppressive due to the high proportion of myeloid cells, such as tumor associated macrophages (TAMs), and, particularly, the M2 phenotype highly represented in GBM [22]. M2 TAMs have pro-tumoral activity through the secretion of immunosuppressive molecules such as transforming growth factor β (TGFβ), interleukin 10 (IL-10), vascular endothelial growth factor (VEGF), or matrix metalloproteinase (MMP)-2 and MMP-9 [23]. TAMs are an active field of research, and are also described to be unpolarized with an M0 phenotype, and therefore would represent an ideal target for reprogramming [24]. GBM cells can also secrete TGFβ that can further induce regulatory T cells (Tregs), which amplifies the immune suppression [25]. The enzyme indoleamine-pyrrole 2,3-dioxygenase (IDO) is another suppressive factor in the GBM TME that will degrade tryptophan, an amino acid needed to promote T cell growth and survival [26]. Taken together, immunological specificities of the CNS, characteristics of the GBM tumor itself, and the TME lead to an unfavorable immune state with sparse T cell infiltration [27,28,29], which can classify GBM as an immunologically cold tumor.

#### 1.1.3. Cellular Pathways

Detailed molecular description of GBM led to the identification of alterations of well-known cellular pathways (such as PI3K, AKT, mTOR, p53, EGFR, IDH, *MET*, FGFR, *BRAF*, and TERT) that could potentially be actionable with tyrosine kinase inhibitors (TKI) or other therapeutics. We will not address the issues of the clinical trials in this review, as it has already been thoroughly done elsewhere [30] and presents no clinical breakthrough. Ongoing trials may still lead to specific approaches for selected patients with GBM. For example, in pleomorphic xanthoastrocytoma, the targeting of *BRAF*^V600^ in patients presenting the mutation led to a survival increase compared to a placebo [31]. Moreover, patients suffering from neurofibromatosis type 1-associated brain tumors could benefit from an *MEK* inhibitor, as reviewed previously [32].

### 1.2. Current Treatment

The standard of care (SOC) treatment for GBM evolved in 2005 with the combined approach, showing the efficacy of radio-chemotherapy with temozolomide (TMZ) after maximum safe surgical removal [33,34]. Since then, no therapeutic improvement could be brought to the patients suffering from GBM except with tumor treating fields (TTFs), which improved survival from 16 months in the maintenance temozolomide group (TMZ) versus 20.9 months for the maintenance TMZ plus a TTF [35]. Still, the TTF approach has not been widely adopted because of a lack of understanding of the underlying mode of action, and the differences between first and second line efficacy [36]. Moreover, the O6-alkylguanine DNA alkyltransferase (MGMT) promoter needs to be methylated for optimal TMZ sensitivity [37]. Inevitably, all GBM will recur, and no effective treatment has been identified in this setting. Bevacizumab, an anti-VEGF Ab improves progression free survival (PFS), but not overall survival (OS) [38], and lomustine is the most used molecule at recurrence in selected patients [39]. The choice of therapy at recurrence is mainly based on the ability of patients to tolerate more chemotherapy, and if cerebral edema is predominant, bevacizumab would be the preferred choice. Still, many clinical trials have attempted to improve SOC with protocol intensification [40,41,42], or to identify new therapies such as tyrosine kinase inhibitors (TKI) [30], but none of them could radically change the clinical course of GBM. As such, we might need to get back to GBM tumor biology to better identify GBM vulnerabilities and strategies to exploit them, such as through synthetic lethality, as identified for *PTEN* deficient gliomas, for example [43]. In the following sections, we will explore different strategies to target the GBM immune specificities mentioned above and tested in clinical trials. We will try to identify potential weaknesses of specific approaches and hypotheses to enhance future research. We will purposefully not list all clinical immunotherapies for GBM, as the readers can refer to previous extensive reviews [44].We will rather select key clinical trials assembled in Table 1.

## 2. Current Knowledge

### 2.1. Immune Checkpoints Inhibitors

Immune checkpoints are key molecules regulating immune activation, some of them being negative regulators [55]. By blocking it with immune checkpoint inhibitors (ICI), immune response can be unleashed. Immune regulatory molecules can be expressed by the tumor cells or immune cells. Ab blocking the PD-1/PD-L1 axis and the CTLA-4 molecule are the most studied and already clinically approved ICI for multiple cancer indications such as melanoma or lung cancer. Recent clinical trials specifically tested their efficacy for GBM. The first phase III study, CheckMate 143, evaluated nivolumab (anti-PD-1) in patients with recurrent GBM [45]. In this study, the control group was treated with bevacizumab, and the primary end point of OS was not improved with ICI. Two other phase III trials, CheckMate 498 [56] and 548, evaluated nivolumab and radiotherapy compared to SOC in primary GBM, either in MGMT unmethylated or methylated, respectively. None of them could identify the efficacy of ICI, but complete data are not yet published. The only significant improvement of OS with ICI was observed in a phase II clinical trial when pembrolizumab was introduced in a neo-adjuvant setting. In this study with recurrent GBM, both groups received pembrolizumab as an adjuvant treatment, but the OS was 13.7 months in the group receiving one injection before surgery, compared to 7.5 months in the control group [47]. Another study also tested neo-adjuvant anti-PD-1 for recurrent GBM, but could not show clinical benefit with an OS of 7.3 months [46]. The impact of the positive neo-adjuvant study is important, as it is the first time we have observed survival impact with ICI in GBM patients. Higher Ag load at the time of therapy initiation might allow broader adaptive immunity activation. Insofar as partial or even subtotal resection leaves less tumor tissue, it is potentially more controllable by the immune system. Moreover, in the recurrent setting, all patients have received TMZ, which has been described to induce hypermutated GBM [57] and could therefore potentiate to ICI. Unfortunately, we do not have data on the TMB of patients, which could impact on the response. However, recent work discredits the idea of better ICI response of hypermutated GBM when it has been induced by previous TMZ treatment [58]. The explanation could be that, if chemotherapy can increase the number of mutations, it will lead to polyclonality, which will not support better immune response [59]. To that matter, case reports have identified hypermutated GBM induced by mismatch repair gene mutations with contrasting response to ICI [60].

As GBM are full of TAM that expresses PD-L1 [22], the use of anti-PD-L1 Ab could also potentially induce antibody-dependent cell-mediated cytotoxicity (ADCC) to further enhance the response. A first study with recurrent GBM combined anti PD-L1 (avelumab) with axitinib, but did not meet its activity threshold of 50% PFS at 6 months [61]. The MEDI4736 study evaluated durvalumab, another anti-PD-L1 Ab, with SOC for primary MGMT unmethylated GBM, and showed OS improvement to 15.7 months, but only when compared with historical controls [62]. Multiple other immune checkpoints exist, such as GITR, TIGIT, CD27, LAG-3, CD137 (or 4-1BB), and CTLA-4, and their blockade was also tested, mostly in association with anti-PD-1, which showed efficacy in pre-clinical glioma mouse models [63,64,65,66,67], but clinical trials are still ongoing. In the end, ICI have already been widely tested for GBM and did not lead to practice changing observations. The highly positive hint of ICI impact on GBM when pembrolizumab was added prior to surgery encourages us to pursue the clinical optimization of ICI therapy. However, as GBM cells present few NeoAg, enhancing the recognition phase, i.e., with vaccination, may be necessary to initiate the immune reaction.

### 2.2. Vaccines

Different types of tumor vaccine exist, and all aim to induce humoral or cellular response against tumor Ags. They can be directed against tumor associated antigens (TAAs), which are host antigens aberrantly expressed on tumor cells, or tumor specific antigens (TSA), which are derived from mutations specific to the tumor cells leading to NeoAgs. Vaccines can either target one or multiple peptides from both categories, and can be administered either simply with an adjuvant, or loaded in DC. Many clinical trials evaluated the efficacy of DC vaccines, but only one was a phase III trial. DCVax-L evaluated a tumor lysate DC vaccine in association with SOC compared to SOC with a placebo for primary GBM [50]. At recurrence, all patients were allowed to receive the DC vaccine, leading to 90% of the patients being treated with the therapy after cross-over. Based on an intention-to-treat analysis, the OS was 23.1 months, and up to 34.7 months for MGMT methylated patients. Unfortunately, the analysis is still preliminary as PFS data are not available, even though it was the primary endpoint, and OS data are also incomplete as the interim analysis includes only 33% of patients [68]. Thus, complete data analysis is needed to draw any conclusion from this trial.

Peptide vaccination is another option exploited, for example, in the IMA-950 trial. In this study, vaccines contained multiple epitopes expressed in GBM samples [69]. This set of synthetic peptides administered with the TLR3 agonist poly-ICLC adjuvant was combined with SOC. The therapy was safe and could elicit immune reaction and reports an OS of 19 months [49]. Results are still preliminary but prove the safety and feasibility of the IMA-950 vaccine. Limitations of peptide vaccination include the HLA restriction and expression, to ensure the exposure of injected epitopes. Moreover, because TAA are expressed by host cells, tolerance could be induced. Along that line, there is a rationale for combining vaccination with ICI, which is currently under evaluation with this vaccine (NCT03665545).

Another approach is to target TSA, as they are not expressed by healthy tissues. Long deletion in the EGFR locus leads to a variant of the EGFR receptor called EGFRvIII. This NeoAg is present in approximately 30% [70] of GBM patients and represents an ideal target for vaccination. ACT-IV, a phase III clinical trial studying the efficacy of peptide vaccination for this particular target, failed to prove efficacy despite encouraging previous data showing immunogenicity [48]. The heterogeneous expression of the target and loss of expression, even in the control arm, were major concerns that can explain the lack of efficacy and question the real oncogenic role of this variant. To circumvent the possibility of Ag loss, the GAPVAC trial aimed at developing a personalized vaccine targeting, at the same time, multiple neoepitopes expressed by the tumor. Due to the time needed for the production of such a vaccine, patients were first treated with a combination of unmutated peptide vaccine, but still matched for their expression. Fifteen patients completed the protocol, and the trial reports an OS of 29 months [71]. In this trial, despite all the mutations analyzed from the 15 patients, only a few of them were predicted to be immunogenic, and none of them could be confirmed to be presented by the tumor cells with mass spectrometry. As a result, a maximum of 2 different NeoAg were included in the TSA vaccination for a minority of patients (4/15), illustrating the limitations of such a technique for tumors with a low mutational load.

In conclusion, if the proof of concept of immunization against GBM is acknowledged, it still does not translate to clear clinical benefit. One particular limitation is the target selection and potential loss of expression, even though multiplying target specificity in the GAPVAC trial was not the solution to dramatically improve efficacy. Combination with ICI, as is already being explored, is promising, as it could enhance the efficacy of the newly activated lymphocytes to drive efficient immune reaction, even further if TAA are used. Still, targeting the TME will be necessary, as it drives important immunosuppression at the tumor site that even fully activated T cells may not bypass.

### 2.3. TME Targeting

As GBM tumors are embedded within an immune hostile environment, its modulation may be necessary to enable better immune responses at the tumor site. One major component of the TME are TAM, which represent up to 50% of the tumor cells [72] and are mostly immunosuppressive, as mentioned before. TAM can be directly targeted through the colony stimulating factor 1 receptor (CSF1R), which is a canonical marker of TAMs. This receptor is necessary for TAM differentiation, proliferation, and activation. Blocking CSF1R can re-educate TAM from M2 to the more anti-tumoral M1 phenotype [73]. Preclinical data with the small molecule BLZ945 are very encouraging [10], and currently tested in association with an anti-PD-1 Ab for solid tumors, including GBM (NCT02829723). Another trial evaluating CSF1R inhibition in association with nivolumab was conducted, but shares no results [74], and a clinical trial evaluating a small molecule PLX3397 inhibiting CSF1R failed to show activity on GBM [75].

Additionally, stimulation of the CD40 pathway can activate TAM to become tumoricidal [76]. In that way, agonistic anti-CD40 stimulation was promising in a pre-clinical model of pancreatic cancer [77,78,79], and is currently being tested as an intra tumoral injection in association with an immunotoxin targeting EGFR [80].

TAM phagocytosis is controlled by multiple layers. If M1 macrophages are considered anti-tumoral and with effective phagocytic functionality, molecules such as CD47 can be expressed by tumor cells and send a “do not eat me” signal to TAM. Therefore, strategies to block this pathway with antagonistic Ab are well described [81]. For human GBM, CD47 is found to be expressed [82], and antagonistic Abs in pre-clinical models show some activity [83]. One of the major issues of CD47 as a target is its expression on physiological red blood cells, but off-tumor effect can be controlled with bi-specific Abs [84]. Still, no clinical trials are currently evaluating anti-CD47 for GBM at this time. Due to their abundance in GBM, there is interest in targeting TAMs, as they perform a broad sampling of the highly heterogeneous tumor, and, because they can cross-present peptides to T cells, they could bridge innate to adaptive immunity [85].

By targeting VEGF, the Ab bevacizumab is also targeting the TME, and is approved in some countries as a second line treatment. As mentioned before, it does not improve OS, only PFS [38], and is currently tested in association with multiple other therapies.

Targeting the TGFβ pathway has also been tested with the small molecule galunisertinib, in monotherapy or in association with lomustine, for recurrent GBM, but no efficacy was described [86]. Other strategies such as the antisense oligonucleotide trabedersen (AP 12009) were tested to inhibit TGFβ signaling [87], but with no clinical efficacy. IDO inhibition is also being evaluating for GBM, supported by encouraging pre-clinical results [64], still, no clinical data are available.

Tregs exerts an immunosuppressive role, and are described as invading GBM. As CTLA-4 is expressed on Tregs, anti-CTLA-4 Ab could potentially induce ADCC of Tregs [88]. Given that IL-2R is essential for Treg function [89], blocking it with the daclizumab Ab, concomitantly with EGFRvIII vaccination, showed a reduction of circulating Tregs in a pilot study [90].

Radiotherapy (RT) is part of the SOC for GBM, but also has interesting immune activity. It can lead to Ag release, and increase CD8 tumor infiltration to further sensitize to ICI therapy in pre-clinical GBM models [63,64]. Furthermore, activation of the CGAS/STING pathway with RT leads to innate immunity activation [91], but careful selection of dose and fraction are needed as they can have contrasting effects on the immune system [92]. For example, RT will lead to CXCL-12 and CSF-1 tumor cell secretion [92], which promotes TAM infiltration and tilts the balance towards pro-tumoral. Moreover, the engulfment of apoptotic cells leads to a tolerance of TAM [85]. Based on this, rational combination with every type of immunotherapy, but particularly ICI and TAM targeting, seems rational, and radiation is often included in current clinical trials.

Intriguingly, cancer associated fibroblasts (CAF) have been identified in GBM patients while they were presumably absent of CNS tumors. Now, some reports describe the presence of CAF with immunosuppressive effects, such as promoting glioma initiating cells growth and TAM polarization towards M2 [93,94]. Moreover, identification of CAF in GBM samples, but not from unaffected brain tissues in autopsy studies, further support their existence in vivo, rather than being only a genomic finding artefact. If CAF’s immunosuppressive function is confirmed, it will certainly be necessary to co-target it with TAM to ensure broader control of the TME. In short, immune-favorable TME is needed for efficient immunotherapy. The complexity of interactions in the TME are being deciphered with a combination of different modalities, but most importantly, evaluation of TME immune state is strongly needed in current and future clinical trials to help orient optimal combinations.

### 2.4. Adoptive Cell Therapy

As host immune response can be difficult to elicit in patients with GBM, engineering an immune activation in autologous T cells can circumvent this issue. Adoptive transfer of circulating autologous T cells, natural killer (NK) cells, or tumor-infiltrating T cells (TILs), expanded and/or activated in vitro before re-infusion, have been developed for cancer immune-therapy and already tested in GBM, but none of these trials led to significant survival improvement. One major issue is the lack of specificity of the infused cells. Even for TIL therapy, we know that only a minority of TILs are tumor specific [95], and if they can be expanded in vitro, they are highly exhausted and poorly functional [96].

CAR (chimeric antigen receptor)-T cells, on the other hand, are developed to specifically target tumor cells. Studies with EGFRvIII targeting CAR-T cells have been conducted showing the feasibility of such technology for GBM [51]. Particularly, CAR-T cells have been found at the tumor site, still with no objective clinical response. A major issue with CAR-T therapy can be off-tumor on-target effect. We reported unexpected CD19 expression in brain mural cells, providing insights on the mechanism of the severe neurotoxicity observed in the clinic [97].

One caveat of single Ag targeting, as identified with the EGFRvIII vaccine, is the loss of expression and the immune escape of tumor cells. To that matter, co-targeting multiple epitopes, either with co-expression on the same CAR-T cell or by combining multiple monovalent CAR-T cells, is currently being explored in other cancers [98], but also in GBM by targeting EGFRvIII, IL13Rα2, HER2, or EphA2 [99]. Pre-clinical studies shows that mathematical models can help us to determine the optimal combination of targets [100], and confirms that co-targeting multiple epitopes with the same CAR-T cell can increase the selectivity and activation of a specific CAR-T, without leading to more exhaustion [101]. Other targets are also in development, such as CD133, which is a cancer stem cell marker. A recent study described the development of anti-CD133 CAR-T cells and their efficacy in preclinical models. We now await clinical trials to see if targeting CD133+ cells would enhance the durable response. In fact, direct lysis of these treatment resistant and self-populating glioma cells could have an impact on tumor control [102]. Still, if glioma initiating cells do not reach consensus [103], the rationale of targeting resistant stem like cells is highly relevant, and will most probably generate interesting data to explore. In the future, conditional activation of CAR-T cells based on multiple targets or sensors with Boolean output based on AND, OR, and NOT coding, could lead to specific activity based on the given environment where the cell is present. The technology has already been described, and needs to be incorporated into clinical investigations [104]. Synthetic biology with inducible T cell programming could address the major caveats of CAR-T cell therapy, and even more for GBM, i.e., (i) immune-related side effects, (ii) potential off-target response, (iii) and conditioned response to the TME, which is particularly decisive in GBM immunotherapy.

Still, if multiplying targets, or conditioning CAR-T cell activation, can reduce immune escape, it will come at high time and money costs that could impact on clinical feasibility. Moreover, tumor cells may still be susceptible to immune escape. Yet, if adoptive T cell therapy can induce immune activation at the tumor site, for particularly cold tumors such as GBM, it could lead to epitope spreading. This could bridge the host adaptive immune system to target other Ags, and, furthermore, be able to adapt to any evolution of the tumor phenotype and sustain the therapeutic efficacy initiated by CAR-T cells. In that way, the combination of CAR-T cell therapy with a systemic immune activation using ICI is a way forward and being already explored [105], even though particular attention to side effects is required with such immune activation.

Another key question to resolve is the optimal route of infusion, especially for GBM, where T cells need a specific integrin, α4β1, to cross the BBB [15]. Intra-cavital infusion post tumor resection has been tested [98], but we lack clear comparison of the infusion route on efficacy in the clinic, even if preclinical data robustly suggest locoregional infusion to be more efficient than systemic delivery [106,107]. In the same way, a study described PAX4 as a key regulator of tumor vascularization, and interfering with its signal improved CAR-T cell influx to the tumor [108].

Another approach could be the targeting of intracellular Ags with TCR engineering, as demonstrated in pediatric diffuse intrinsic pontine glioma, where the substitution from lysine (K) to methionine (M) at position 27 of histone 3 variant 3 (H3.3) is present in approximately 70% of cases. To that matter, TCR transduced T cells can be developed to specifically target this mutation, as suggested in this promising pre-clinical study [109].

If adoptive cell therapy, and particularly CAR-T cell therapy, presents an unprecedented therapeutic possibility, it also comes with a high complexity. It may take time to master optimal target selection and safety. Yet, if the feasibility of CAR-T cell therapy for GBM is validated, its clinical efficacy still needs to be proven.

### 2.5. Oncolytic Viruses

If the potential cytolytic effect of such therapy was initially aimed, the ability to convert “cold” tumors to “hot” could be even more clinically relevant. The PVSRIPO trial studied a modified polio virus infused intratumorally at recurrence and showed the safety of administration [53]. Another trial studied an oncolytic adenovirus, DNX-2401, which showed local replication and hints at local immune activation [54]. Both trials report long term responders, and further clinical trials are awaited to potentially confirm significant survival efficacy. We refer the interested readers to the reference [110] for a detailed description of oncolytic viruses for GBM.

## 3. Discussion

SOC for GBM has not been improved since 2005, and multiple clinical trials to optimize combined RT and TMZ struggled to further increase survival [40,41]. Moreover, SOC can lead to irreversible toxicity, such as neurological deficits due to surgery, neuro-cognitive impairments with radiotherapy, and systemic toxicity induced by alkylation or nitrosourea [111]. As mentioned above, even though deep characterization of molecular alterations of GBM identified potential targets that have already proved their efficacy for other cancer indications, none of the TKI evaluated could lead to significant impact [30]. Drug penetrance was once mentioned as a limitation, but even osimertinib, which has optimized BBB penetrance, did not improve survival [112]. Moreover, techniques exist to enhance drug delivery by focally modulating BBB permeability, such as high intensity focused ultrasound [113], and could address the potential limitations of delivery. For immunotherapy, and particularly ICI, significant responses against brain metastasis [3] seem to indicate that immune response is achievable in the CNS. Though, intrinsic GBM characteristics, such as low TMB and TME specificities, make it more difficult to tackle. As mentioned above, all clinical trials evaluating ICI efficacy failed to increase survival, with the exception of the neoadjuvant pembrolizumab [47], but more mature data are needed to confirm this highly promising efficacy signal. It is unlikely that mono-immunotherapy would drive a strong response, and combination trials to increase immune response are needed, as are for other non-CNS, primary ICI refractory malignancies.

**Figure 1:**
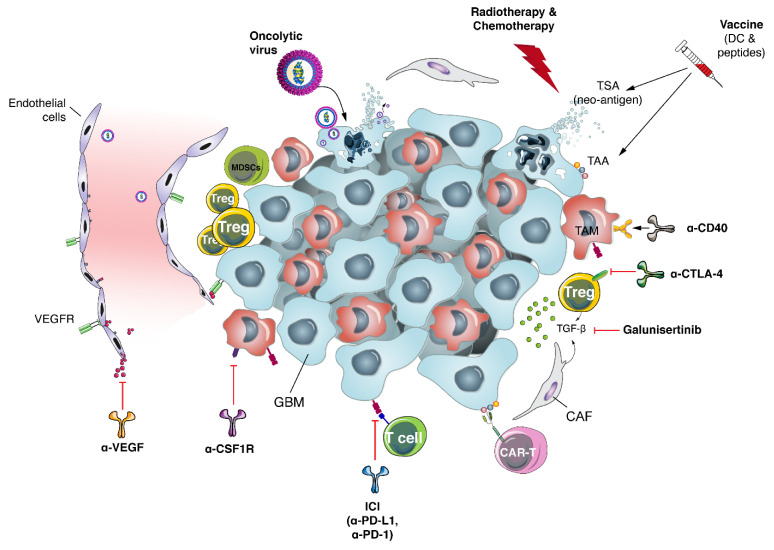
Key cell components of a glioblastoma tumor, including potential targets and therapies. Arrows are indicative of activation/induction (black) or inhibition (red).

As all therapeutic combinations cannot be tested in clinical trials, detailed evaluation of the available data and the use of rationally selected pre-clinical models will empower future clinical trials. Molecular knowledge of GBM has exponentially increased within the past 10 years, and current classification of such tumors can rely almost only on genomic alterations [5]. Moreover, the use of cutting-edge technologies such as CRISPR/Cas9 mutation screening, pushed forward even our understanding of relevant genetic alterations of human GBM [114]. Single-cell analysis of all components of GBM tumors also gives us a sharp knowledge of multiple components of such tumors [115,116]. We are still far from understanding the multiple components of highly heterogeneous tumors such as GBM and their implication in tumor growth or immune response. More studies and deeper characterization of the phenotype and functionality of myeloid cells seems key to understanding tumor dynamics, as it has been shown for other tumors such as lung cancer [117,118]. In the Figure 1, we depict key GBM TME components and potential strategies to generate better anti-tumoral immune response.

Multiple pre-clinical models exist, and their different characteristics can influence results. Up to now, the most used model is GL261, which presents highly immunogenic characteristics [8], a central feature to studying intra-CNS immune reaction but leading to an overestimation of the response to immunotherapy. Other models such as SB28 [8] or spontaneous GBM models [9] demonstrate immunological features closer to human GBM, and will empower our knowledge to initiate immune response for highly resistant tumors such as GBM. For in vitro studies, human brain organoids better reproduce cell organization, and can add an extra level of relevance with more precise cell interactions [119].

Current SOC also impacts on immunotherapy response. As we saw above, RT has the opposite impact depending on its application. On the other hand, chemotherapy can lead to the accumulation of stochastic mutations. Specifically, TMZ has been described as inducing hypermutation in 17% of treated patients in one study [57]. In theory, higher TMB should enhance response to immunotherapy, as it leads to the potential generation of more NeoAg, but it also comes with a downside, as randomly acquired mutations will create multiple subclones [59], therefore diluting the potential immune response and limiting response to ICI [58]. Neoadjuvant clinical trials give us the unique opportunity of exploring the effect of therapeutic interventions, particularly in brain tumors with limited accessibility for biopsies. Biomarkers and immuno-monitoring for predicting and following therapeutic response are a constant field of research in current oncology, and the rise of circulating tumor DNA follow-up might become a standard in the next decade. For GBM, such approach is already studied, but with yet no clinical application [120].

A common clinical situation in GBM is brain edema management, which is most commonly controlled with immuno-suppressive steroids. Bypassing the need for steroids is therefore of particular interest when immunotherapy is envisaged. As dexamethasone is described as decreasing T cell activation, it will therefore limit the immunotherapy activity [121]. However, this postulate needs to be nuanced, as one research paper studying the effect of dexamethasone on human T cells, and also with GL261 implanted mice, described a blockade of naïve T cell proliferation and differentiation that can be partially rescued with anti-CTLA-4 treatment, but not anti-PD-1 [122]. The Ipi-Glio clinical trial is specifically addressing the efficacy of the combination of ipilimumab to adjuvant TMZ after SOC, and will therefore give us insight on this specific blockade [123]. Moreover, another study identified anti-PD-1 therapy to be limited by corticosteroid use in mice with extra CNS tumors, but no differences were observed with GL261-luc orthotopic implantation, therefore tempering the limiting effect of dexamethasone for intra-CNS tumors [124]. In an experimental autoimmune encephalomyelitis model, dexamethasone has been shown to restore BBB function and limit bystander T cell infiltration, while keeping Ag specific T cells untouched [125]. Additionally, corticosteroids can also stimulate MerTK in TAM, which has anti-tumoral activity [126]. In the end, corticosteroids mainly appear to limit T cell activation, and strategies to rescue immune activation, or alternatives to their use, should be explored. One alternative to corticosteroids for the management of refractory brain edema is bevacizumab [127], which should not interfere with the immune response. Additionally, complement activation is also liked to brain edema, and inhibitors are being studied [128]. Still, only preclinical data are available in the context of stroke or brain hemorrhage [129], but, such as with bevacizumab, this approach could help us limit steroid use, and potentially favors immune activation.

Radioisotope-bound Abs, or Ab drug conjugates, also represent an innovative approach to selectively targeting tumor cells. Both therapies could rationally be combined with other immunotherapeutic approaches, as they can lead to better immune activation through immunogenic cell death with less systemic side effects. For prostate cancer, lutetium-177-labeled anti-PSMA monoclonal antibody has been developed and is currently being explored clinically [130]. Still, these techniques are tied to the limitation of target selection, and need more robust clinical data.

Ongoing clinical trials are a major opportunity to collect crucial data needed to elucidate current open questions. To that matter, rational design based on laboratory data and monitoring is key to not only evaluating the efficacy of a new protocol, but to ensuring the high value of data and the guidance of future studies. As such, we pledge for academic initiated trials ensuring broader application and the availability of the collected data.

## 4. Conclusions

Altogether, multiple hints are pointing towards immune activation with different immunotherapeutic strategies. Recent findings are shedding light on the potential immunotherapy impact on GBM, but more clinical trials are needed to find the optimal combination to finally bring survival benefit to patients with GBM. To that matter, meticulous data collection during clinical trials and rational pre-clinical studies are needed to empower our knowledge and understanding of immunotherapy for GBM, and to pave the way for the next decade dedicated to bringing immunotherapy efficacy to GBM.

## Figures and Tables

**Table 1:** Key clinical studies with different immunotherapeutic modalities for GBM.

Clinical Trial	Phase	Indication	Treatment	Control	Sequence	Outcome	Ref
Immune Checkpoints Inhibitors							
CheckMate 143 NCT02017717	III	R GBM	Nivolumab	Bevacizumab	At recurrence Nivo monotherapy	No impact on OS	2020 [45]
CheckMate 498 NCT02617589	III	P GBM MGMT u	Nivolumab RT	TMZ + RT	Nivo + RT, then Nivo	No impact on OS	
CheckMate 548 NCT02667587	II	P GBM MGMT m	Nivolumab TMZ + RT	TMZ + RT	TMZ + RT, then TMZ ± Nivo	No impact on OS *	
Neo-nivo NCT02550249	II	P GBM R GBM	Nivolumab	None	Nivo, surgery, then Nivo (+TMZ + RT if primary)	OS: 7.3 m *	2019 [46]
NCT02852655	II	R GBM	Pembrolizumab Neoadj + adj	Pembrolizumab adj	±Pembro, then Surgery, then Pembro	OS: 13.7 vs. 7.5 m *	2019 [47]
**Vaccines**							
ACT-IV NCT01480479	III	P GBM EGFRvIII+	Peptide vaccine	Placebo	Surgery, RT + TMZ, then vaccine	No impact on OS	2017 [48]
IMA-950 NCT01920191	I/II	P GBM HLA-A2+	TAA peptide vaccine	None	Surgery, RT + TMZ, then vaccine	OS: 19 mo *	2019 [49]
DCVax-L NCT00045968	III	P GBM	Tumor lysate DC vaccine	Placebo	Surgery, RT + TMZ, then vaccine	OS: 23.1 mo *	2018 [50]
**CAR-T Cells**							
NCT02209376	I	R GBM EGFRvIII+	EGFRvIII CAR-T cell (2nd gen)	None	CAR-T infusion at progression	OS: 8 mo *	2017 [51]
NCT01454596	I	R GBM EGFRvIII+	EGFRvIII CAR-T cell (3rd gen)	None	Lymphodepleting chemotherapy, then CAR-T infusion + IL-2 support	OS: 6.9 mo *	2019 [52]
**Oncolytic Virus**							
NCT01491893	II	R GBM	PVSRIPO	None	IT administration of PVSRIPO at recurrence	OS: 12.5 mo *	2018 [53]
NCT02798406	II	R Glioma malignant	DNX-2401	None	IT administration of DNX-2401 ± surgical resection	OS: 9.5 mo *	2018 [54]

Abbreviations: TAA, tumor-associated antigen; P GBM, primary glioblastoma; R GBM, recurrent glioblastoma; HLA, human leukocyte antigen; OS, overall survival; MGMTu, MGMG unmethylated; MGMTm, MGMG methylated; Nivo, nivolumab; Pembro, pembrolizumab; RT, radiotherapy; TMZ, temozolomide; CAR-T, chimeric antigen receptor T cell; DC, dendritic cell; IT, intra-tumoral; adj, adjuvant; * OS data should be taken with reserve as it was not the primary endpoint of the study.

## Data Availability

Not applicable.

## References

[1] Ostrom Q.T., Gittleman H., Liao P., Vecchione-Koval T., Wolinsky Y., Kruchko C., Barnholtz-Sloan J.S. (2017). CBTRUS Statistical Report: Primary brain and other central nervous system tumors diagnosed in the United States in 2010-2014. Neuro-Oncology.

[2] Carson M.J., Doose J.M., Melchior B., Schmid C.D., Ploix C.C. (2006). CNS immune privilege: Hiding in plain sight. Immunol. Rev..

[3] Tran T.T., Jilaveanu L.B., Omuro A., Chiang V.L., Huttner A., Kluger H.M. (2019). Complications associated with immunotherapy for brain metastases. Curr. Opin. Neurol..

[4] Louis D.N., Ohgaki H., Wiestler O.D., Cavenee W.K. (2016). WHO Classification of Tumours of the Central Nervous System.

[5] Weller M., van den Bent M., Preusser M., Le Rhun E., Tonn J.C., Minniti G., Bendszus M., Balana C., Chinot O., Dirven L. (2021). EANO guidelines on the diagnosis and treatment of diffuse gliomas of adulthood. Nat. Rev. Clin. Oncol..

[6] Verhaak R.G., Hoadley K.A., Purdom E., Wang V., Qi Y., Wilkerson M.D., Miller C.R., Ding L., Golub T., Mesirov J.P. (2010). Integrated genomic analysis identifies clinically relevant subtypes of glioblastoma characterized by abnormalities in PDGFRA, IDH1, EGFR, and NF1. Cancer Cell.

[7] Patel A.P., Tirosh I., Trombetta J.J., Shalek A.K., Gillespie S.M., Wakimoto H., Cahill D.P., Nahed B.V., Curry W.T., Martuza R.L. (2014). Single-cell RNA-seq highlights intratumoral heterogeneity in primary glioblastoma. Science.

[8] Genoud V., Marinari E., Nikolaev S.I., Castle J.C., Bukur V., Dietrich P.Y., Okada H., Walker P.R. (2018). Responsiveness to anti-PD-1 and anti-CTLA-4 immune checkpoint blockade in SB28 and GL261 mouse glioma models. Oncoimmunology.

[9] Hambardzumyan D., Amankulor N.M., Helmy K.Y., Becher O.J., Holland E.C. (2009). Modeling Adult Gliomas Using RCAS/t-va Technology. Transl. Oncol..

[10] Pyonteck S.M., Akkari L., Schuhmacher A.J., Bowman R.L., Sevenich L., Quail D.F., Olson O.C., Quick M.L., Huse J.T., Teijeiro V. (2013). CSF-1R inhibition alters macrophage polarization and blocks glioma progression. Nat. Med..

[11] Karimi S., Zuccato J.A., Mamatjan Y., Mansouri S., Suppiah S., Nassiri F., Diamandis P., Munoz D.G., Aldape K.D., Zadeh G. (2019). The central nervous system tumor methylation classifier changes neuro-oncology practice for challenging brain tumor diagnoses and directly impacts patient care. Clin. Epigenetics.

[12] Schumacher T.N., Schreiber R.D. (2015). Neoantigens in cancer immunotherapy. Science.

[13] Wu A., Wiesner S., Xiao J., Ericson K., Chen W., Hall W.A., Low W.C., Ohlfest J.R. (2007). Expression of MHC I and NK ligands on human CD133+ glioma cells: Possible targets of immunotherapy. J. Neurooncol..

[14] Chen D.S., Mellman I. (2013). Oncology meets immunology: The cancer-immunity cycle. Immunity.

[15] Calzascia T., Masson F., Di Berardino-Besson W., Contassot E., Wilmotte R., Aurrand-Lions M., Ruegg C., Dietrich P.Y., Walker P.R. (2005). Homing phenotypes of tumor-specific CD8 T cells are predetermined at the tumor site by crosspresenting APCs. Immunity.

[16] Stoll G., Jander S., Schroeter M. (2002). Detrimental and beneficial effects of injury-induced inflammation and cytokine expression in the nervous system. Adv. Exp. Med. Biol..

[17] Arvanitis C.D., Ferraro G.B., Jain R.K. (2020). The blood-brain barrier and blood-tumour barrier in brain tumours and metastases. Nat. Rev. Cancer.

[18] Dijkers E.C., Oude Munnink T.H., Kosterink J.G., Brouwers A.H., Jager P.L., de Jong J.R., van Dongen G.A., Schroder C.P., Lub-de Hooge M.N., de Vries E.G. (2010). Biodistribution of 89Zr-trastuzumab and PET imaging of HER2-positive lesions in patients with metastatic breast cancer. Clin. Pharmacol. Ther..

[19] Louveau A., Smirnov I., Keyes T.J., Eccles J.D., Rouhani S.J., Peske J.D., Derecki N.C., Castle D., Mandell J.W., Lee K.S. (2015). Structural and functional features of central nervous system lymphatic vessels. Nature.

[20] Da Mesquita S., Fu Z., Kipnis J. (2018). The Meningeal Lymphatic System: A New Player in Neurophysiology. Neuron.

[21] Poon C.C., Sarkar S., Yong V.W., Kelly J.J.P. (2017). Glioblastoma-associated microglia and macrophages: Targets for therapies to improve prognosis. Brain.

[22] Komohara Y., Ohnishi K., Kuratsu J., Takeya M. (2008). Possible involvement of the M2 anti-inflammatory macrophage phenotype in growth of human gliomas. J. Pathol..

[23] Mantovani A., Sozzani S., Locati M., Allavena P., Sica A. (2002). Macrophage polarization: Tumor-associated macrophages as a paradigm for polarized M2 mononuclear phagocytes. Trends Immunol..

[24] Kowal J., Kornete M., Joyce J.A. (2019). Re-education of macrophages as a therapeutic strategy in cancer. Immunotherapy.

[25] Schwyzer M., Fontana A. (1985). Partial purification and biochemical characterization of a T cell suppressor factor produced by human glioblastoma cells. J. Immunol..

[26] Scott K.E., Cleveland J.L. (2016). Lactate Wreaks Havoc on Tumor-Infiltrating T and NK Cells. Cell Metab..

[27] Rutledge W.C., Kong J., Gao J., Gutman D.A., Cooper L.A., Appin C., Park Y., Scarpace L., Mikkelsen T., Cohen M.L. (2013). Tumor-infiltrating lymphocytes in glioblastoma are associated with specific genomic alterations and related to transcriptional class. Clin. Cancer Res..

[28] Berghoff A.S., Kiesel B., Widhalm G., Rajky O., Ricken G., Wohrer A., Dieckmann K., Filipits M., Brandstetter A., Weller M. (2015). Programmed death ligand 1 expression and tumor-infiltrating lymphocytes in glioblastoma. Neuro-Oncology.

[29] Han S., Zhang C., Li Q., Dong J., Liu Y., Huang Y., Jiang T., Wu A. (2014). Tumour-infiltrating CD4(+) and CD8(+) lymphocytes as predictors of clinical outcome in glioma. Br. J. Cancer.

[30] Le Rhun E., Preusser M., Roth P., Reardon D.A., van den Bent M., Wen P., Reifenberger G., Weller M. (2019). Molecular targeted therapy of glioblastoma. Cancer Treat. Rev..

[31] Kaley T., Touat M., Subbiah V., Hollebecque A., Rodon J., Lockhart A.C., Keedy V., Bielle F., Hofheinz R.D., Joly F. (2018). *BRAF* Inhibition in *BRAF*(V600)-Mutant Gliomas: Results From the VE-BASKET Study. J. Clin. Oncol..

[32] Klesse L.J., Jordan J.T., Radtke H.B., Rosser T., Schorry E., Ullrich N., Viskochil D., Knight P., Plotkin S.R., Yohay K. (2020). The Use of *MEK* Inhibitors in Neurofibromatosis Type 1-Associated Tumors and Management of Toxicities. Oncologist.

[33] Stupp R., Mason W.P., van den Bent M.J., Weller M., Fisher B., Taphoorn M.J., Belanger K., Brandes A.A., Marosi C., Bogdahn U. (2005). Radiotherapy plus concomitant and adjuvant temozolomide for glioblastoma. N. Engl. J. Med..

[34] Mirimanoff R.O., Gorlia T., Mason W., Van den Bent M.J., Kortmann R.D., Fisher B., Reni M., Brandes A.A., Curschmann J., Villa S. (2006). Radiotherapy and temozolomide for newly diagnosed glioblastoma: Recursive partitioning analysis of the EORTC 26981/22981-NCIC CE3 phase III randomized trial. J. Clin. Oncol..

[35] Stupp R., Taillibert S., Kanner A., Read W., Steinberg D., Lhermitte B., Toms S., Idbaih A., Ahluwalia M.S., Fink K. (2017). Effect of Tumor-Treating Fields Plus Maintenance Temozolomide vs Maintenance Temozolomide Alone on Survival in Patients With Glioblastoma: A Randomized Clinical Trial. JAMA.

[36] Wick W. (2016). TTFields: Where does all the skepticism come from?. Neuro-Oncology.

[37] Hegi M.E., Diserens A.C., Gorlia T., Hamou M.F., de Tribolet N., Weller M., Kros J.M., Hainfellner J.A., Mason W., Mariani L. (2005). MGMT gene silencing and benefit from temozolomide in glioblastoma. N. Engl. J. Med..

[38] Wick W., Gorlia T., Bendszus M., Taphoorn M., Sahm F., Harting I., Brandes A.A., Taal W., Domont J., Idbaih A. (2017). Lomustine and Bevacizumab in Progressive Glioblastoma. N. Engl. J. Med..

[39] Weller M., Le Rhun E. (2020). How did lomustine become standard of care in recurrent glioblastoma?. Cancer Treat Rev..

[40] Gilbert M.R., Wang M., Aldape K.D., Stupp R., Hegi M.E., Jaeckle K.A., Armstrong T.S., Wefel J.S., Won M., Blumenthal D.T. (2013). Dose-dense temozolomide for newly diagnosed glioblastoma: A randomized phase III clinical trial. J. Clin. Oncol..

[41] Balana C., Vaz M.A., Manuel Sepulveda J., Mesia C., Del Barco S., Pineda E., Munoz-Langa J., Estival A., de Las Penas R., Fuster J. (2020). A phase II randomized, multicenter, open-label trial of continuing adjuvant temozolomide beyond 6 cycles in patients with glioblastoma (GEINO 14-01). Neuro-Oncology.

[42] Herrlinger U., Tzaridis T., Mack F., Steinbach J.P., Schlegel U., Sabel M., Hau P., Kortmann R.D., Krex D., Grauer O. (2019). Lomustine-temozolomide combination therapy versus standard temozolomide therapy in patients with newly diagnosed glioblastoma with methylated MGMT promoter (CeTeG/NOA-09): A randomised, open-label, phase 3 trial. Lancet.

[43] Chen P., Zhao D., Li J., Liang X., Li J., Chang A., Henry V.K., Lan Z., Spring D.J., Rao G. (2019). Symbiotic Macrophage-Glioma Cell Interactions Reveal Synthetic Lethality in PTEN-Null Glioma. Cancer Cell.

[44] Weenink B., French P.J., Sillevis Smitt P.A.E., Debets R., Geurts M. (2020). Immunotherapy in Glioblastoma: Current Shortcomings and Future Perspectives. Cancers.

[45] Reardon D.A., Omuro A., Brandes A.A., Rieger J., Wick A., Sepulveda J., Phuphanich S., de Souza P., Ahluwalia M.S., Lim M. (2017). OS10.3 Randomized Phase 3 Study Evaluating the Efficacy and Safety of Nivolumab vs Bevacizumab in Patients With Recurrent Glioblastoma: CheckMate 143. Neuro-Oncology.

[46] Schalper K.A., Rodriguez-Ruiz M.E., Diez-Valle R., Lopez-Janeiro A., Porciuncula A., Idoate M.A., Inoges S., de Andrea C., Lopez-Diaz de Cerio A., Tejada S. (2019). Neoadjuvant nivolumab modifies the tumor immune microenvironment in resectable glioblastoma. Nat. Med..

[47] Cloughesy T.F., Mochizuki A.Y., Orpilla J.R., Hugo W., Lee A.H., Davidson T.B., Wang A.C., Ellingson B.M., Rytlewski J.A., Sanders C.M. (2019). Neoadjuvant anti-PD-1 immunotherapy promotes a survival benefit with intratumoral and systemic immune responses in recurrent glioblastoma. Nat. Med..

[48] Weller M., Butowski N., Tran D.D., Recht L.D., Lim M., Hirte H., Ashby L., Mechtler L., Goldlust S.A., Iwamoto F. (2017). Rindopepimut with temozolomide for patients with newly diagnosed, EGFRvIII-expressing glioblastoma (ACT IV): A randomised, double-blind, international phase 3 trial. Lancet Oncol..

[49] Migliorini D., Dutoit V., Allard M., Grandjean Hallez N., Marinari E., Widmer V., Philippin G., Corlazzoli F., Gustave R., Kreutzfeldt M. (2019). Phase I/II trial testing safety and immunogenicity of the multipeptide IMA950/poly-ICLC vaccine in newly diagnosed adult malignant astrocytoma patients. Neuro-Oncology.

[50] Liau L.M., Ashkan K., Tran D.D., Campian J.L., Trusheim J.E., Cobbs C.S., Heth J.A., Salacz M., Taylor S., D’Andre S.D. (2018). First results on survival from a large Phase 3 clinical trial of an autologous dendritic cell vaccine in newly diagnosed glioblastoma. J. Transl. Med..

[51] O’Rourke D.M., Nasrallah M.P., Desai A., Melenhorst J.J., Mansfield K., Morrissette J.J.D., Martinez-Lage M., Brem S., Maloney E., Shen A. (2017). A single dose of peripherally infused EGFRvIII-directed CAR T cells mediates antigen loss and induces adaptive resistance in patients with recurrent glioblastoma. Sci. Transl. Med..

[52] Goff S.L., Morgan R.A., Yang J.C., Sherry R.M., Robbins P.F., Restifo N.P., Feldman S.A., Lu Y.C., Lu L., Zheng Z. (2019). Pilot Trial of Adoptive Transfer of Chimeric Antigen Receptor-transduced T Cells Targeting EGFRvIII in Patients With Glioblastoma. J. Immunother..

[53] Desjardins A., Gromeier M., Herndon J.E., Beaubier N., Bolognesi D.P., Friedman A.H., Friedman H.S., McSherry F., Muscat A.M., Nair S. (2018). Recurrent Glioblastoma Treated with Recombinant Poliovirus. N. Engl. J. Med..

[54] Lang F.F., Conrad C., Gomez-Manzano C., Yung W.K.A., Sawaya R., Weinberg J.S., Prabhu S.S., Rao G., Fuller G.N., Aldape K.D. (2018). Phase I Study of DNX-2401 (Delta-24-RGD) Oncolytic Adenovirus: Replication and Immunotherapeutic Effects in Recurrent Malignant Glioma. J. Clin. Oncol..

[55] Pardoll D.M. (2012). The blockade of immune checkpoints in cancer immunotherapy. Nat. Rev. Cancer.

[56] Sampson J.H., Omuro A.M.P., Preusser M., Lim M., Butowski N.A., Cloughesy T.F., Strauss L.C., Latek R.R., Paliwal P., Weller M. (2016). A Randomized, Phase 3, Open-Label Study of Nivolumab versus Temozolomide (TMZ) in Combination with Radiotherapy (RT) in Adult Patients (pts) with Newly Diagnosed, O-6-Methylguanine DNA Methyltransferase (MGMT)-Unmethylated Glioblastoma (GBM): CheckMate-498.

[57] Wang J., Cazzato E., Ladewig E., Frattini V., Rosenbloom D.I., Zairis S., Abate F., Liu Z., Elliott O., Shin Y.J. (2016). Clonal evolution of glioblastoma under therapy. Nat. Genet..

[58] Touat M., Li Y.Y., Boynton A.N., Spurr L.F., Iorgulescu J.B., Bohrson C.L., Cortes-Ciriano I., Birzu C., Geduldig J.E., Pelton K. (2020). Mechanisms and therapeutic implications of hypermutation in gliomas. Nature.

[59] McGranahan N., Furness A.J., Rosenthal R., Ramskov S., Lyngaa R., Saini S.K., Jamal-Hanjani M., Wilson G.A., Birkbak N.J., Hiley C.T. (2016). Clonal neoantigens elicit T cell immunoreactivity and sensitivity to immune checkpoint blockade. Science.

[60] Bouffet E., Larouche V., Campbell B.B., Merico D., de Borja R., Aronson M., Durno C., Krueger J., Cabric V., Ramaswamy V. (2016). Immune Checkpoint Inhibition for Hypermutant Glioblastoma Multiforme Resulting From Germline Biallelic Mismatch Repair Deficiency. J. Clin. Oncol..

[61] Neyns B., Ben Salama L., Awada G., De Cremer J., Schwarze J.K., Seynaeve L., Du Four S., Fischbuch L., Vanbinst A.-M., Everaert H. (2019). GLIAVAX: A Stratified Phase II Clinical Trial of Avelumab and Axitinib in Patients with Recurrent Glioblastoma.

[62] Reardon D.A., Kaley T.J., Dietrich J., Clarke J.L., Dunn G., Lim M., Cloughesy T.F., Gan H.K., Park A.J., Schwarzenberger P. (2019). Phase II Study to Evaluate Safety and Efficacy of MEDI4736 (Durvalumab)+ Radiotherapy in Patients with Newly Diagnosed Unmethylated MGMT Glioblastoma (New Unmeth GBM).

[63] Kim J.E., Patel M.A., Mangraviti A., Kim E.S., Theodros D., Velarde E., Liu A., Sankey E.W., Tam A., Xu H. (2017). Combination Therapy with Anti-PD-1, Anti-TIM-3, and Focal Radiation Results in Regression of Murine Gliomas. Clin. Cancer Res..

[64] Ladomersky E., Zhai L., Lenzen A., Lauing K.L., Qian J., Scholtens D.M., Gritsina G., Sun X., Liu Y., Yu F. (2018). IDO1 Inhibition Synergizes with Radiation and PD-1 Blockade to Durably Increase Survival Against Advanced Glioblastoma. Clin. Cancer Res..

[65] Wainwright D.A., Balyasnikova I.V., Han Y., Lesniak M.S. (2011). The expression of BST2 in human and experimental mouse brain tumors. Exp. Mol. Pathol..

[66] Saha D., Martuza R.L., Rabkin S.D. (2018). Oncolytic herpes simplex virus immunovirotherapy in combination with immune checkpoint blockade to treat glioblastoma. Immunotherapy.

[67] Patel M.A., Kim J.E., Theodros D., Tam A., Velarde E., Kochel C.M., Francica B., Nirschl T.R., Ghasemzadeh A., Mathios D. (2016). Agonist anti-GITR monoclonal antibody and stereotactic radiation induce immune-mediated survival advantage in murine intracranial glioma. J. Immunother. Cancer.

[68] Wick W., van den Bent M.J. (2018). First results on the DCVax phase III trial: Raising more questions than providing answers. Neuro-Oncology.

[69] Dutoit V., Herold-Mende C., Hilf N., Schoor O., Beckhove P., Bucher J., Dorsch K., Flohr S., Fritsche J., Lewandrowski P. (2012). Exploiting the glioblastoma peptidome to discover novel tumour-associated antigens for immunotherapy. Brain.

[70] Congdon K.L., Gedeon P.C., Suryadevara C.M., Caruso H.G., Cooper L.J., Heimberger A.B., Sampson J.H. (2014). Epidermal growth factor receptor and variant III targeted immunotherapy. Neuro-Oncology.

[71] Hilf N., Kuttruff-Coqui S., Frenzel K., Bukur V., Stevanovic S., Gouttefangeas C., Platten M., Tabatabai G., Dutoit V., van der Burg S.H. (2019). Actively personalized vaccination trial for newly diagnosed glioblastoma. Nature.

[72] Rossi M.L., Hughes J.T., Esiri M.M., Coakham H.B., Brownell D.B. (1987). Immunohistological study of mononuclear cell infiltrate in malignant gliomas. Acta Neuropathol..

[73] Cannarile M.A., Weisser M., Jacob W., Jegg A.M., Ries C.H., Ruttinger D. (2017). Colony-stimulating factor 1 receptor (CSF1R) inhibitors in cancer therapy. J. Immunother. Cancer.

[74] Brahmer J., Rasco D., Chen M., Masteller E., Qazi I., Rogers S., Sankar N., Sikorski R., Hambleton J., Hodi F.S. (2016). Abstract B143: A Phase 1a/1b Study of FPA008 in Combination with Nivolumab in Patients with Selected Advanced Cancers.

[75] Butowski N., Colman H., De Groot J.F., Omuro A.M., Nayak L., Wen P.Y., Cloughesy T.F., Marimuthu A., Haidar S., Perry A. (2016). Orally administered colony stimulating factor 1 receptor inhibitor PLX3397 in recurrent glioblastoma: An Ivy Foundation Early Phase Clinical Trials Consortium phase II study. Neuro-Oncology.

[76] Vonderheide R.H., Glennie M.J. (2013). Agonistic CD40 antibodies and cancer therapy. Clin. Cancer Res..

[77] Winograd R., Byrne K.T., Evans R.A., Odorizzi P.M., Meyer A.R., Bajor D.L., Clendenin C., Stanger B.Z., Furth E.E., Wherry E.J. (2015). Induction of T-cell Immunity Overcomes Complete Resistance to PD-1 and CTLA-4 Blockade and Improves Survival in Pancreatic Carcinoma. Cancer Immunol. Res..

[78] O’Hara M.H., O’Reilly E.M., Varadhachary G., Wolff R.A., Wainberg Z.A., Ko A.H., Fisher G., Rahma O., Lyman J.P., Cabanski C.R. (2021). CD40 agonistic monoclonal antibody APX005M (sotigalimab) and chemotherapy, with or without nivolumab, for the treatment of metastatic pancreatic adenocarcinoma: An open-label, multicentre, phase 1b study. Lancet Oncol..

[79] Beatty G.L., Chiorean E.G., Fishman M.P., Saboury B., Teitelbaum U.R., Sun W., Huhn R.D., Song W., Li D., Sharp L.L. (2011). CD40 agonists alter tumor stroma and show efficacy against pancreatic carcinoma in mice and humans. Science.

[80] Phase 1 Trial of D2C7-IT in Combination with 2141-V11 for Recurrent Malignant Glioma. https://ClinicalTrials.gov/show/NCT04547777.

[81] Kim D., Wang J., Willingham S.B., Martin R., Wernig G., Weissman I.L. (2012). Anti-CD47 antibodies promote phagocytosis and inhibit the growth of human myeloma cells. Leukemia.

[82] Li F., Lv B., Liu Y., Hua T., Han J., Sun C., Xu L., Zhang Z., Feng Z., Cai Y. (2018). Blocking the CD47-SIRPalpha axis by delivery of anti-CD47 antibody induces antitumor effects in glioma and glioma stem cells. Oncoimmunology.

[83] Gholamin S., Mitra S.S., Feroze A.H., Liu J., Kahn S.A., Zhang M., Esparza R., Richard C., Ramaswamy V., Remke M. (2017). Disrupting the CD47-SIRPalpha anti-phagocytic axis by a humanized anti-CD47 antibody is an efficacious treatment for malignant pediatric brain tumors. Sci. Transl. Med..

[84] Dheilly E., Moine V., Broyer L., Salgado-Pires S., Johnson Z., Papaioannou A., Cons L., Calloud S., Majocchi S., Nelson R. (2017). Selective Blockade of the Ubiquitous Checkpoint Receptor CD47 Is Enabled by Dual-Targeting Bispecific Antibodies. Mol. Ther..

[85] Lecoultre M., Dutoit V., Walker P.R. (2020). Phagocytic function of tumor-associated macrophages as a key determinant of tumor progression control: A review. J. Immunother. Cancer.

[86] Brandes A.A., Carpentier A.F., Kesari S., Sepulveda-Sanchez J.M., Wheeler H.R., Chinot O., Cher L., Steinbach J.P., Capper D., Specenier P. (2016). A Phase II randomized study of galunisertib monotherapy or galunisertib plus lomustine compared with lomustine monotherapy in patients with recurrent glioblastoma. Neuro-Oncology.

[87] Bogdahn U., Hau P., Stockhammer G., Venkataramana N.K., Mahapatra A.K., Suri A., Balasubramaniam A., Nair S., Oliushine V., Parfenov V. (2011). Targeted therapy for high-grade glioma with the TGF-beta2 inhibitor trabedersen: Results of a randomized and controlled phase IIb study. Neuro-Oncology.

[88] Arce Vargas F., Furness A.J.S., Litchfield K., Joshi K., Rosenthal R., Ghorani E., Solomon I., Lesko M.H., Ruef N., Roddie C. (2018). Fc Effector Function Contributes to the Activity of Human Anti-CTLA-4 Antibodies. Cancer Cell.

[89] Cheng G., Yu A., Malek T.R. (2011). T-cell tolerance and the multi-functional role of IL-2R signaling in T-regulatory cells. Immunol. Rev..

[90] Sampson J.H., Schmittling R.J., Archer G.E., Congdon K.L., Nair S.K., Reap E.A., Desjardins A., Friedman A.H., Friedman H.S., Herndon J.E. (2012). A pilot study of IL-2Ralpha blockade during lymphopenia depletes regulatory T-cells and correlates with enhanced immunity in patients with glioblastoma. PLoS ONE.

[91] Vanpouille-Box C., Alard A., Aryankalayil M.J., Sarfraz Y., Diamond J.M., Schneider R.J., Inghirami G., Coleman C.N., Formenti S.C., Demaria S. (2017). DNA exonuclease Trex1 regulates radiotherapy-induced tumour immunogenicity. Nat. Commun..

[92] Rodriguez-Ruiz M.E., Vitale I., Harrington K.J., Melero I., Galluzzi L. (2020). Immunological impact of cell death signaling driven by radiation on the tumor microenvironment. Nat. Immunol..

[93] Rick J., Nguyen A., Chandra A., Wadhwa H., Shah S., Wang L., Lau D., Safaee M., Ordaz A., Yagnik G. (2019). TMIC-22. Identification of cancer-associated fibroblasts in glioblastoma and defining their protumoral effects. Neuro-Oncology.

[94] Liu T., Zhou L., Li D., Andl T., Zhang Y. (2019). Cancer-Associated Fibroblasts Build and Secure the Tumor Microenvironment. Front. Cell Dev. Biol..

[95] Simoni Y., Becht E., Fehlings M., Loh C.Y., Koo S.L., Teng K.W.W., Yeong J.P.S., Nahar R., Zhang T., Kared H. (2018). Bystander CD8(+) T cells are abundant and phenotypically distinct in human tumour infiltrates. Nature.

[96] Rohaan M.W., van den Berg J.H., Kvistborg P., Haanen J. (2018). Adoptive transfer of tumor-infiltrating lymphocytes in melanoma: A viable treatment option. J. Immunother. Cancer.

[97] Parker K.R., Migliorini D., Perkey E., Yost K.E., Bhaduri A., Bagga P., Haris M., Wilson N.E., Liu F., Gabunia K. (2020). Single-Cell Analyses Identify Brain Mural Cells Expressing CD19 as Potential Off-Tumor Targets for CAR-T Immunotherapies. Cell.

[98] Migliorini D., Dietrich P.Y., Stupp R., Linette G.P., Posey A.D., June C.H. (2018). CAR T-Cell Therapies in Glioblastoma: A First Look. Clin. Cancer Res..

[99] Han X., Wang Y., Wei J., Han W. (2019). Multi-antigen-targeted chimeric antigen receptor T cells for cancer therapy. J. Hematol. Oncol..

[100] Hegde M., Corder A., Chow K.K., Mukherjee M., Ashoori A., Kew Y., Zhang Y.J., Baskin D.S., Merchant F.A., Brawley V.S. (2013). Combinational targeting offsets antigen escape and enhances effector functions of adoptively transferred T cells in glioblastoma. Mol. Ther..

[101] Hegde M., Mukherjee M., Grada Z., Pignata A., Landi D., Navai S.A., Wakefield A., Fousek K., Bielamowicz K., Chow K.K. (2016). Tandem CAR T cells targeting HER2 and IL13Ralpha2 mitigate tumor antigen escape. J. Clin. Investig..

[102] Vora P., Venugopal C., Salim S.K., Tatari N., Bakhshinyan D., Singh M., Seyfrid M., Upreti D., Rentas S., Wong N. (2020). The Rational Development of CD133-Targeting Immunotherapies for Glioblastoma. Cell Stem Cell.

[103] Saito N., Hirai N., Aoki K., Sato S., Suzuki R., Hiramoto Y., Fujita S., Nakayama H., Hayashi M., Sakurai T. (2019). Genetic and Lineage Classification of Glioma-Initiating Cells Identifies a Clinically Relevant Glioblastoma Model. Cancers.

[104] Williams J.Z., Allen G.M., Shah D., Sterin I.S., Kim K.H., Garcia V.P., Shavey G.E., Yu W., Puig-Saus C., Tsoi J. (2020). Precise T cell recognition programs designed by transcriptionally linking multiple receptors. Science.

[105] Grosser R., Cherkassky L., Chintala N., Adusumilli P.S. (2019). Combination Immunotherapy with CAR T Cells and Checkpoint Blockade for the Treatment of Solid Tumors. Cancer Cell.

[106] Brown C.E., Aguilar B., Starr R., Yang X., Chang W.C., Weng L., Chang B., Sarkissian A., Brito A., Sanchez J.F. (2018). Optimization of IL13Ralpha2-Targeted Chimeric Antigen Receptor T Cells for Improved Anti-tumor Efficacy against Glioblastoma. Mol. Ther..

[107] Theruvath J., Sotillo E., Mount C.W., Graef C.M., Delaidelli A., Heitzeneder S., Labanieh L., Dhingra S., Leruste A., Majzner R.G. (2020). Locoregionally administered B7-H3-targeted CAR T cells for treatment of atypical teratoid/rhabdoid tumors. Nat. Med..

[108] Ma W., Wang Y., Zhang R., Yang F., Zhang D., Huang M., Zhang L., Dorsey J.F., Binder Z.A., O’Rourke D.M. (2020). Targeting PAK4 to reprogram the vascular microenvironment and improve CAR-T immunotherapy for glioblastoma. Nat. Cancer.

[109] Chheda Z.S., Kohanbash G., Okada K., Jahan N., Sidney J., Pecoraro M., Yang X., Carrera D.A., Downey K.M., Shrivastav S. (2018). Novel and shared neoantigen derived from histone 3 variant H3.3K27M mutation for glioma T cell therapy. J. Exp. Med..

[110] Sostoa J., Dutoit V., Migliorini D. (2020). Oncolytic Viruses as a Platform for the Treatment of Malignant Brain Tumors. Int J. Mol. Sci..

[111] Alemany M., Velasco R., Simó M., Bruna J. (2020). Late effects of cancer treatment: Consequences for long-term brain cancer survivors. Neuro Oncol. Pract..

[112] Abousaud M., Faroqui N., Hsu F.-C., Lesser G., Strowd R., Ramkissoon S., Kwatra M., Houston K.S., Carter A., DeTroye A. (2020). DDRE-17. Initial clinical experience using osimertinib in patients with recurrent malignant gliomas with egfr alterations. Neuro-Oncology.

[113] Appelboom G., Detappe A., LoPresti M., Kunjachan S., Mitrasinovic S., Goldman S., Chang S.D., Tillement O. (2016). Stereotactic modulation of blood-brain barrier permeability to enhance drug delivery. Neuro-Oncology.

[114] MacLeod G., Bozek D.A., Rajakulendran N., Monteiro V., Ahmadi M., Steinhart Z., Kushida M.M., Yu H., Coutinho F.J., Cavalli F.M.G. (2019). Genome-Wide CRISPR-Cas9 Screens Expose Genetic Vulnerabilities and Mechanisms of Temozolomide Sensitivity in Glioblastoma Stem Cells. Cell Rep..

[115] Darmanis S., Sloan S.A., Croote D., Mignardi M., Chernikova S., Samghababi P., Zhang Y., Neff N., Kowarsky M., Caneda C. (2017). Single-Cell RNA-Seq Analysis of Infiltrating Neoplastic Cells at the Migrating Front. of Human Glioblastoma. Cell Rep..

[116] Mathewson N.D., Ashenberg O., Tirosh I., Gritsch S., Perez E.M., Marx S., Jerby-Arnon L., Chanoch-Myers R., Hara T., Richman A.R. (2021). Inhibitory CD161 receptor identified in glioma-infiltrating T cells by single-cell analysis. Cell.

[117] Zilionis R., Engblom C., Pfirschke C., Savova V., Zemmour D., Saatcioglu H.D., Krishnan I., Maroni G., Meyerovitz C.V., Kerwin C.M. (2019). Single-Cell Transcriptomics of Human and Mouse Lung Cancers Reveals Conserved Myeloid Populations across Individuals and Species. Immunity.

[118] Klemm F., Maas R.R., Bowman R.L., Kornete M., Soukup K., Nassiri S., Brouland J.P., Iacobuzio-Donahue C.A., Brennan C., Tabar V. (2020). Interrogation of the Microenvironmental Landscape in Brain Tumors Reveals Disease-Specific Alterations of Immune Cells. Cell.

[119] Cosset E., Locatelli M., Marteyn A., Lescuyer P., Dall Antonia F., Mor F.M., Preynat-Seauve O., Stoppini L., Tieng V. (2019). Human Neural Organoids for Studying Brain Cancer and Neurodegenerative Diseases. J. Vis. Exp..

[120] Le Rhun E., Seoane J., Salzet M., Soffietti R., Weller M. (2020). Liquid biopsies for diagnosing and monitoring primary tumors of the central nervous system. Cancer Lett..

[121] Pitter K.L., Tamagno I., Alikhanyan K., Hosni-Ahmed A., Pattwell S.S., Donnola S., Dai C., Ozawa T., Chang M., Chan T.A. (2016). Corticosteroids compromise survival in glioblastoma. Brain.

[122] Giles A.J., Hutchinson M.N.D., Sonnemann H.M., Jung J., Fecci P.E., Ratnam N.M., Zhang W., Song H., Bailey R., Davis D. (2018). Dexamethasone-induced immunosuppression: Mechanisms and implications for immunotherapy. J. Immunother. Cancer.

[123] Brown N.F., Ng S.M., Brooks C., Coutts T., Holmes J., Roberts C., Elhussein L., Hoskin P., Maughan T., Blagden S. (2020). A phase II open label, randomised study of ipilimumab with temozolomide versus temozolomide alone after surgery and chemoradiotherapy in patients with recently diagnosed glioblastoma: The Ipi-Glio trial protocol. BMC Cancer.

[124] Maxwell R., Luksik A.S., Garzon-Muvdi T., Hung A.L., Kim E.S., Wu A., Xia Y., Belcaid Z., Gorelick N., Choi J. (2018). Contrasting impact of corticosteroids on anti-PD-1 immunotherapy efficacy for tumor histologies located within or outside the central nervous system. Oncoimmunology.

[125] Wust S., van den Brandt J., Tischner D., Kleiman A., Tuckermann J.P., Gold R., Luhder F., Reichardt H.M. (2008). Peripheral T cells are the therapeutic targets of glucocorticoids in experimental autoimmune encephalomyelitis. J. Immunol..

[126] Zagorska A., Traves P.G., Lew E.D., Dransfield I., Lemke G. (2014). Diversification of TAM receptor tyrosine kinase function. Nat. Immunol..

[127] Meng X., Zhao R., Shen G., Dong D., Ding L., Wu S. (2017). Efficacy and safety of bevacizumab treatment for refractory brain edema: Case report. Medicine.

[128] Gong Y., Xi G.H., Keep R.F., Hoff J.T., Hua Y. (2005). Complement inhibition attenuates brain edema and neurological deficits induced by thrombin. Acta Neurochir. Suppl..

[129] Weiss E., Dhir T., Collett A., Reola M., Kaplan M., Minimo C., Omert L., Leung P. (2020). Effect of complement C1-esterase inhibitor on brain edema and inflammation after mild traumatic brain injury in an animal model. Clin. Exp. Emerg. Med..

[130] Tagawa S.T., Vallabhajosula S., Christos P.J., Jhanwar Y.S., Batra J.S., Lam L., Osborne J., Beltran H., Molina A.M., Goldsmith S.J. (2019). Phase 1/2 study of fractionated dose lutetium-177-labeled anti-prostate-specific membrane antigen monoclonal antibody J591 ((177) Lu-J591) for metastatic castration-resistant prostate cancer. Cancer.

